# Novel Polyester Amide Membranes Containing Biquinoline Units and Complex with Cu(I): Synthesis, Characterization, and Approbation for n-Heptane Isolation from Organic Mixtures

**DOI:** 10.3390/polym12030645

**Published:** 2020-03-12

**Authors:** Alexandra Pulyalina, Ilya Faykov, Vera Nesterova, Mikhail Goikhman, Irina Podeshvo, Nairi Loretsyan, Alexander Novikov, Iosif Gofman, Alexander Toikka, Galina Polotskaya

**Affiliations:** 1 Saint Petersburg State University, Institute of Chemistry, Universitetskiy pr. 26, 198504 Saint Petersburg, Russia; st022544@student.spbu.ru (I.F.); st049110@student.spbu.ru (V.N.); a.s.novikov@spbu.ru (A.N.); a.toikka@spbu.ru (A.T.); polotskaya@hq.macro.ru (G.P.); 2 Institute of Macromolecular Compounds, Russian Academy of Sciences, Bolshoy pr. 31, 199004 Saint Petersburg, Russia; goikhman@hq.macro.ru (M.G.); podeshvo@hq.macro.ru (I.P.); lorecyan@yandex.ru (N.L.); gofman@imc.macro.ru (I.G.)

**Keywords:** polyester amide, metal–polymer complex, membranes, pervaporation, *n*-heptane isolation

## Abstract

The wide possibilities of designing a chemical structure and creating complexes with transition metals make polymers of heteroaromatic structure interesting objects, from both scientific and practical aspects. In this work, modern biquinoline-containing polymers, namely polyester amide (PEA) and its metal–polymer complex (PEA–Cu(I)), were synthesized and used to form dense flat membranes. A comparative study of their morphology, same physical properties (density, free volume, and contact angles), and thermomechanical characteristics was carried out. The transport properties of the modern membranes were studied during pervaporation, to solve a problem of *n*-heptane isolation from its binary mixtures with thiophene and methanol. It was shown that only the PEA membrane is selective for the separation of thiophene impurities from the mixture with *n*-heptane. In pervaporation of methanol/*n*-heptane mixture, the РЕА–Cu(I) membrane exhibits significantly higher pervaporation separation index, as compared with that of the РЕА membrane.

## 1. Introduction

Polymers of heteroaromatic structure are among the most promising modern materials due to their high thermal and chemical stability, as well as their good mechanical and film-forming properties. Wide opportunities for designing their chemical structure have attracted much attention from researchers in various fields [1,2,3,4,5]. Complexes of transition metals with polymer ligands are an interesting object from both scientific and practical viewpoints [6]. Metal–polymer complexes can be obtained on the basis of heteroaromatic polymers whose backbone was functionalized by units containing biquinoline groups capable of forming coordination bonds with transition metals, particularly copper(I) salts. The chemical structure of such metal–polymer complexes can be defined as pseudo-crosslinked systems with high-strength deformation properties and significant thermal stability, which determine their operational characteristics [7,8,9].

Earlier, we investigated a polyamic acid containing 6,6′-dimethylamine-2,2′-biquinoline fragments in the backbone and metal–polymer complex with Cu(I) on its basis. It was shown that these polyheteroarylenes are promising membrane materials for separation of the methanol–hexane mixture by pervaporation [10]. At the same time, it is known that the physicochemical and transport properties can be efficiently controlled by changing the chemical structure of a polymer and the position of a functional group in the biquinoline fragment [9,10]. 

In the present work, modern biquinoline-containing polymers, namely polyester amide (PEA) and its metal–polymer complex are under the study. Unlike the macromolecular ligands investigated by Polotskaya et al. in [10], novel polymers do not contain carboxyl groups; in this case, the biquinoline fragment is introduced into the polymer chain at 7,7′ positions. Such changes in the chemical structure enhance the flexibility of the polymer chain due to an increase in the number of possible conformations and the absence of polyelectrolyte effects. This, in turn, should lead to a change in the operational characteristics of both the initial PEA and its metal–polymer complex [11,12,13]. It should be noted that the biquinoline ability to form complexes with metals is rather limited. Complexes of biquinoline with ruthenium(III), ruthenium(II), osmium(III), rhodium(III), platinum(III), cobalt(II), cadmium(II), and others are not very stable [14]. At the same time, biquinoline complexes with copper(I) are among the most stable, as the stability constant is equal to 9–10. That is why metal–polymer complexes with Cu(I) were used to obtain membranes. The PEA and PEA–Cu(I) were tested as membrane materials for the process of *n*-heptane separation from organic mixtures.

Moreover, *n*-Heptane is a standard substance for determining detonation resistance or octane level of motor and aviation gasolines, since the octane level of *n*-heptane equals zero by definition. Despite the fact that *n*-heptane is quite widespread in nature as a component of most petroleums, it is very difficult to obtain a standard *n*-heptane. An actual task for chemical and petroleum-refining industries is separation of *n*-heptane from its mixtures with methanol and thiophene.

Gasoline desulfurization is essential because sulfur-containing impurities significantly reduce fuel quality, as well as worsen the environmental situation due to emissions of sulfur oxides in the surrounding environment. The main sulfur-containing impurity in hydrocarbons extracted from the Earth is thiophene; therefore, in this work, a model gasoline consisting of a binary mixture of thiophene and *n*-heptane is considered. As membrane material for removal of thiophene from gasoline, different polymers such as polyphenylene oxide, polyphosphazene, polyvinylidene fluoride, polyhedral oligomeric silsesquioxane POSS, polysulfone, and polyether-block-amide Pebax, have been used [15,16,17,18,19,20,21,22]. 

Various alcohols and ethers are used as oxygenated additives for unleaded gasoline to increase octane level. Methanol has a high oxygen content, high flammability limit, and high burning rate, which allows it to improve some characteristics of the gasoline engine and to reduce emissions into the environment [23,24].

The extraction of methanol from its mixture with alkanes is one of the tasks of the petrochemical industry. The problems of application of such a common method as distillation are connected with the existence of azeotropes [25]. For examples, methanol forms azeotropic mixture with heptane at 101.33 kPa, 46 wt% of alcohol [26]. Therefore, the simple distillation is unsuitable process for this purpose and methods of azeotropic, and extractive distillation should be applied [27]. On the other hand, these processes are energy-intensive, and the using of other methods is more desirable approach. Another industrial alternative method is liquid–liquid extraction [28,29,30], but it is necessary to choose optimal entrainer for effective selection and carrying out regeneration of individual components. At present, pervaporation is a promising membrane technology in separating azeotropic mixtures, solutions with similar boiling points, thermally sensitive compounds, and organic–organic mixtures, as well as in removing dilute organics from aqueous solutions [31,32].

Thus, the tasks of this work included the synthesis of modern biquinoline-containing polyester amide PEA and its metal–polymer complex PEA–Cu(I), the preparation of dense flat membranes, and the study on their structure, thermomechanical properties, and physical properties, as well as approbation for *n*-heptane isolation from its binary mixtures with methanol and thiophene by pervaporation.

## 2. Materials and Methods 

### 2.1. Materials 

Chloral hydrate, hydroxylamine hydrochloride, *m*-toluidine, sulfuric acid, acetoine, sulfolane, selenium dioxide, copper powder, potassium hydroxide, nickel diformate, ammonium formate, formic acid, *N*-methylpyrrolidone (NMP), and propylene oxide were purchased from Sigma-Aldrich and used without additional purification. Other reagents and solvents were purified before use. The 4,4′-Diaminodiphenyl ether was purified by recrystallization from ethyl alcohol (*T*_m_ = 190 °C), and thionyl chloride was purified by simple distillation (*T*_b_ = 78 °C).

#### 2.1.1. Synthesis of Novel Bifunctional Monomeric Ligand 2,2′-Biquinoline-7,7′-Diyldimethanamine (1)

The 2,2′-biquinoline-7,7′-dimethylenediamine was obtained via a multistep process. In the first step, 7,7′-dimethyl-2,2′- biquinoline-4,4′-dicarboxylic acid was obtained by Pfitzinger reaction, using 6-methylisatin and acetoin. Next, dicarboxylic acid was decarboxylated at 300 °C, in the presence of a copper metal powder, to obtain 7,7′-dimethyl-2,2′-biquinoline. The oxidation of methyl groups to aldehyde groups was carried out with selenium dioxide, and the corresponding diformyl derivative 2,2′-biquinoline-7,7′-dicarbaldehyde was obtained. In the final stage, 2,2′-biquinoline-7,7′-diyldimethanamine (1) was synthesized by the Leuckart–Wallach reaction [33].

#### 2.1.2. Synthesis of Terephthaloyl-bis(3-Methoxy-4-oxybenzoic) Acid Dichloride (2)

The synthesis of terephthaloyl-bis(3-methoxy-4-hydroxybenzoic) acid dichloride was performed in accordance with the procedure described in [34].

#### 2.1.3. Synthesis of Polyesteramide with Biquinoline Units in the Main Chain (PEA) (4)

In a flask equipped with a stirrer, 0.08 mmol (0.16 g) of an 2,2′-biquinoline-7,7′-diyldimethanamine (1), 0.02 mmol (0.0628 g) of 4,4′-diaminodiphenyl ether (2), and 6.46 mL of NMP were loaded. The resulting mixture was stirred until full dissolution of diamines and then cooled to −15 °C. To the cooled solution, 0.103 mmol (0.5181 g) of terephthaloylbis (3-methoxy-4-hydroxybenzoic) acid dichloride (3) was added. The suspension was stirred at −15 °C for 30 min, after which the cooling bath was removed, 0.1 mL of propylene oxide was added, and the mixture was stirred at room temperature until a viscous, transparent solution was formed. Then, stirring was continued for another 4 h [35]. 

The PEA–Cu(I) metal–polymer complex (5) was obtained by mixing solutions of PEA and copper(I) chloride (CuCl) in NMP; the molar ratio between CuCl and biquinolyne units in the polymer was 1:2. The interaction occurs at room temperature upon stirring of the dilute solution. 

### 2.2. Membrane Preparation 

Dense flat membranes were obtained by casting 10 wt% polymer solutions of PEA or PEA–Cu(I) in NMP on a glass plate, by solvent evaporation, and by drying in vacuum at 90 °C, to constant weight. The thickness of the obtained membrane was 29–46 μm, and the error of measurements was ± 0.1 μm.

### 2.3. Membrane Characterization

Membrane morphology was studied by scanning electron microscope SEM Zeiss SUPRA 55VP (Carl Zeiss AG, Oberkochen, Germany). Before tests, a graphite layer of 20 nm in thickness was coated on the sample surface by cathode sputtering, using the Quorum 150 (Great Britain) setup.

ATR-FTIR spectra of the membranes were recorded on IR-Fourier spectrometer Iraffinity-1 (Shimadzu, Kyoto, Japan), with a resolution of 1 cm^−1^ within the range of 4000–650 cm^−1^, at ambient temperature (25 °C).

The membrane density (ρ) was measured by hydrostatic weighing method, using a laboratory-made unit. The mixture of isopropyl alcohol (ρ = 0.7851 g/cm^3^) and carbon tetrachloride (ρ = 1.594 g/cm^3^) was used to equilibrate the specimens at 20 °C. The samples of 0.05–0.10 g were used; the error of measurements was ± 0.0001 g cm^‒3^.

The free-volume (*V_f_* ) of the polymer film was calculated by the following equation [36]:

(1) $$V_{f}=V_{0}-1.3V_{w}$$

where *V_0_* = 1/*ρ* is the polymer specific volume, and *V_w_* is the van der Waals volume of the repeat unit calculated by Bondi’s method [37]. The fractional free volume (*FFV*) of the film was calculated by the following equation:

(2) $$FFV=V_{f}/V_{0}$$

The contact angles of water and ethanol on the polymer-film surface were measured at room temperature and atmospheric pressure, using Drop Shape Analyzer DSA 10 (KRÜSS, Hamburg, Germany). Surface tension of water and ethanol at 20 °C are equal to 72.4 and 21.4 mN/m, respectively [38]. Data on measured contact angles were used to calculate membrane surface tension (σs) via the Owens–Wendt method [39,40].

Mechanical properties of the membranes were examined at room temperature via an AG-100kNX Plus universal mechanical test system (Shimadzu), using the uniaxial extension mode. Strip-like samples with the dimensions of 2 × 30 mm were stretched at a rate of 10 mm/min, according to ASTM D638 requirements. The Young’s modulus (*E*), the yield stress (*σ_y_*), the break stress (*σ_b_*), and the ultimate deformation (*ε_b_*) were calculated by using the instrument software.

The temperatures of physical transitions were determined by the thermomechanical method, using a TMA 402 F1 Hyperion thermal analyzer (NETZSCH, Selb, Germany). The tests were carried out with the heating rate of 5 °C/min.

The thermal stability of the materials was investigated, using a DTG-60 thermogravimetric analyzer (Shimadzu). Samples (~5 mg) were heated up to 500 °C, at a rate of 5 °C/min. The experiments were carried out in air. 

### 2.4. Computational Details

The full geometry optimization of model structure was carried out at the DFT level of theory, using the M06-2X functional [41], with the help of the Gaussian-09 program package [42]. No symmetry restrictions were applied during the geometry optimization procedure. The standard 6-31G* basis sets were used for all atoms. The Hessian matrix was calculated analytically for the obtained optimized model structure in order to prove the location of correct minima on the potential energy surface (no imaginary frequencies). The molecular surface electrostatic potential was plotted by using the Chemcraft program [43]. 

### 2.5. Sorption Study

Sorption experiments were performed by immersing membrane samples into individual liquid (*n*-heptane, methanol, and thiophene), at atmospheric pressure and ~20 °C. At certain intervals, the samples were removed and weighed on an analytical balance with an accuracy of ±10^−4^ g. The experiment continued until the constant weight of the swollen sample when the sorption equilibrium was reached (about 30 days). The sorption degree (*S*) was calculated by the equation below:

(3) $$S=[(m_{s}-m_{d})/m_{d}]\cdot 100\%,$$

where *m_s_* is the weight of a swollen membrane upon equilibrium state, and *m_d_* is the weight of a dry membrane.

Data on membrane sorption and density were used to calculate the volume fraction of polymer in swollen membrane (φ_2_):

(4) $$\varphi_{2}=1/\left(1+\frac{\rho_{2}}{\rho_{1}}\cdot S\right),$$

where ρ_1_ and ρ_2_ are a liquid and a polymer densities, respectively. 

The interaction of polymer and a liquid was characterized using the Flory–Huggins theory [44]. The interaction parameter (χ’) was calculated via the following formula [45]:

(5) χ′=−[ln(1−φ2)+φ2]φ22

### 2.6. Pervaporation Test

Pervaporation experiments with binary mixtures were performed, using lab-scale apparatus, with stirring at 20 °C. The effective area of the membrane sample was approximately 14.8 cm^2^. Downstream pressure below 10^−2^ mm Hg was maintained with vacuum pump MD 1C (Vacuubrand GMBH, Wertheim, Germany). The permeate was collected into a trap immersed in liquid nitrogen and weighted with the analytical balance. The composition of feed and product was analyzed by a gas chromatograph Chromatec–Crystal 5000.2 (Chromatec, Yoshkar-Ola, Russia) with a thermal conductivity detector.

The flux through membrane (*J*) was determined as an amount of liquid penetrated through membrane area per unit time.

(6) J=M/S·t

where *M* is the weight of permeate, *S* is the membrane area in contact with feed, and *t* is the operating time.

To compare membranes with different thicknesses (*l*), which varied from 29 to 46 μm, the value of normalized total flux (*J_n_*) was used. *J_n_* is the total flux through membrane with 20 μm thickness, calculated as follows:

*J_n_* = *J* × *l* / 20 (7)

The separation factor *α_i/j_* was defined according to the following equation [46]:

(8) $$\alpha_{i/j}=\left(Y_{i}/Y_{j}\right)/\left(X_{i}/X_{j}\right),$$

where subscripts *i* and *j* refer to separable liquids; *Y* and *X* are the weight fractions of components in the permeate and the feed, respectively. 

Pervaporation separation index (*PSI*) was calculated as follows [47]:

(9) PSI=J·(α−1)

## 3. Results and Discussion

### 3.1. Synthesis of PEA to PEA–Cu(I)

**Figure 1:**
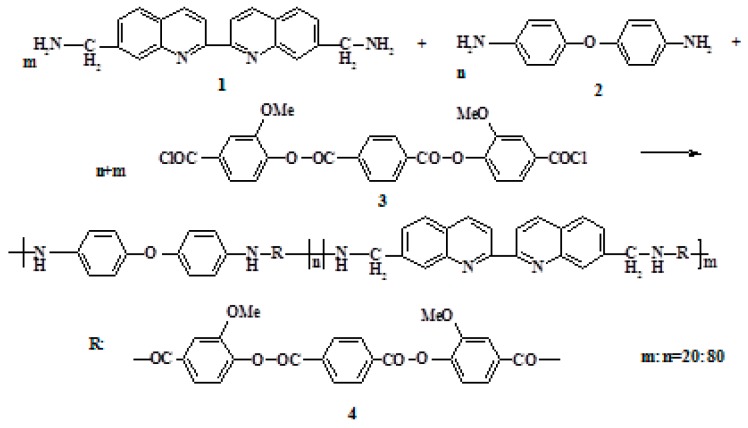
Scheme of PEA synthesis.

The 2,2′-biquinoline-7,7′-diyldimethanamine, 4,4′-diaminodiphenyl ether, and terephthaloylbis (3-methoxy-4-hydroxybenzoic) acid dichloride were used in low-temperature polycondensation, to synthesize a new polyester amide (PEA) containing biquinoline units in the backbone (Figure 1).

**Figure 2:**
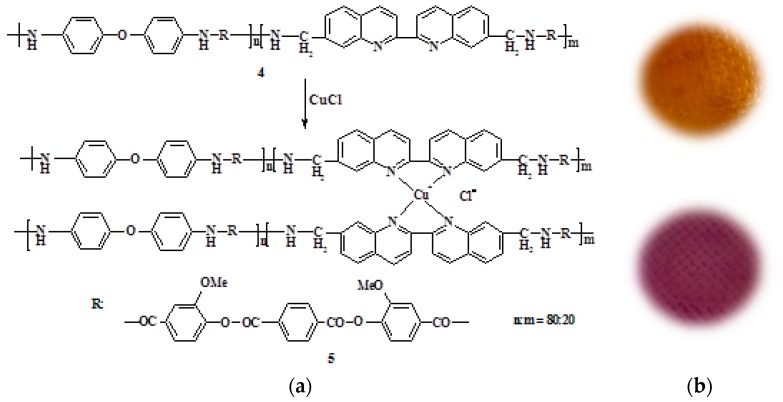
(**a**) Scheme of PEA (**4**) transformation into PEA–Cu(I) (**5**); (**b**) photos of corresponding membrane samples.

By the interaction of the obtained PEA with copper(I) chloride in the NMP solution, the corresponding metal–polymer complex PEA–Cu(I) was prepared. Figure 2 shows the scheme of PEA transformation into PEA–Cu(I) and the photos of corresponding membrane samples showing the color change in this case. The transformation of PEA to PEA–Cu(I) is accompanied by a color change and an increase in the viscosity of the polymer solution. These facts testified to the formation of the metal−polymer complex.

**Figure 3:**
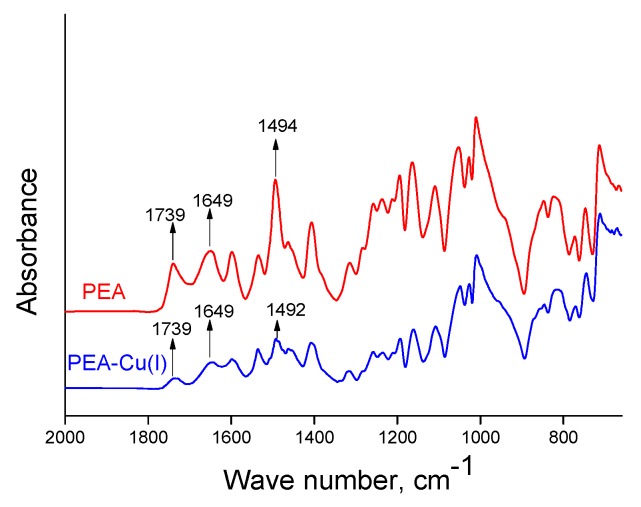
ATR-FTIR spectra of PEA and PEA–Cu(I) membranes.

The chemical structures of obtained polymers were confirmed by IR spectroscopy. Transformation of PEA into PEA–Cu(I) is not accompanied by any obvious changes in ATR-FTIR spectra of the films (Figure 3). The bands at 1649 cm^−1^ correspond to the amide groups of the polymers in both spectra [48]. The bands at 1739 cm^−1^ belong to aromatic ester groups (ν_(C=O)_), which are contained in polyester amide, as well as in the metal–polymer complex. The bands assigned to biquinoline units appear at 1494 and 1492 cm^−1^ for PEA and PEA–Cu(I), respectively.

### 3.2. Membrane Structure

**Figure 4:**
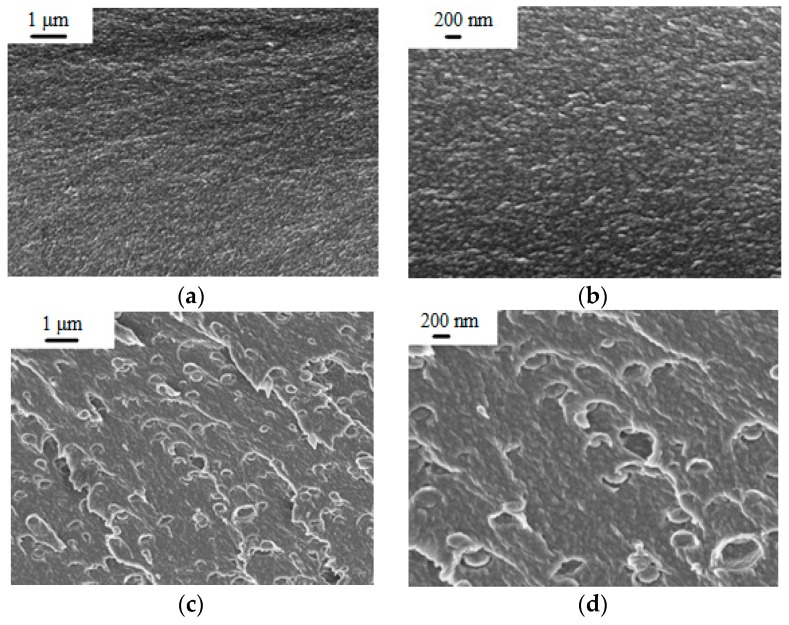
SEM images of membrane cross-section: (**a**,**b**) PEA and (**c**,**d**) PEA–Cu(I).

The PEA and PEA–Cu(I) solutions in NMP were used to obtain dense film membranes by solvent evaporation. To study morphology of the membranes, scanning electron microscopy (SEM) was used. Figure 4 shows cross-section images of PEA and the PEA–Cu(I) membrane with low (1 µm) and high (200 nm) magnification. Both membranes have a dense, homogeneous, defect-free structure. The introduction of Cu(I) ions into the polyester amide contributes to a change in the PEA morphology. In the PEA–Cu(I) membrane, small elements of a supramolecular structure with a diameter up to 300 nm appear. It can arise as a result of rearrangement of the PEA structure during the formation of metal–polymer complexes with copper and removing residual solvent during membranes’ fabrication from a more viscous solution of PEA–Cu(I).

### 3.3. Physical and Mechanical Properties

The density of polymer membranes was determined by the technique of hydrostatic weighing; the values of van der Waals volume of the elementary unit (*V_w_*), free volume (*V_f_*), and fractional free volume (*FFV*) were calculated for PEA and PEA–Cu(I) films, according to the Askadsky method [49]; the results are presented in Table 1. The density of PEA increases after formation of complex with the Cu(I), while the fractional free volume also increases. This fact is unusual for these parameters (density and *FFV*) and can be explained as follows. It is known that the *FFV* depends on the stiffness of the polymer chain, its volume, and the strength of the intermolecular interaction [50,51]. Metal–polymer complex PEA–Cu(I) contains free volume elements caused by loose packing of the polymer chains and additional free volume elements fixed in the polymer due to loss of fragmentary mobility; in our case, this is due to coordination around Cu(I). The presence of a larger free volume in membrane structure of PEA–Cu(I) will probably contribute to a more facilitated transport of penetrants through the polymer matrix.

To characterize the membrane surfaces, contact angles of water and ethanol on the surface of PEA and PEA–Cu(I) films were measured. As can be seen from the data of Table 2, the formation of the metal–polymer complex slightly changes the surface characteristics of the polymer membrane, making the surface of the PEA–Cu(I) a little more hydrophobic, impacting the sorption of molecules on the membrane surface.

Mechanical characteristics, such as Young’s modulus (*E*), yield stress (*σ_y_*), break stress (*σ_b_*), and ultimate deformation (*ε_b_*), were measured at room temperature, under the uniaxial extension. Table 3 shows mechanical and thermal properties of PEA and PEA–Cu(I) membranes. As can be seen, there is a noticeable increase in the Young’s modulus of the PEA–Cu(I) sample, as compared with that of PEA. This effect testifies to the formation of a system of intermolecular crosslinks in the PEA–Cu(I) membranes. 

**Figure 5:**
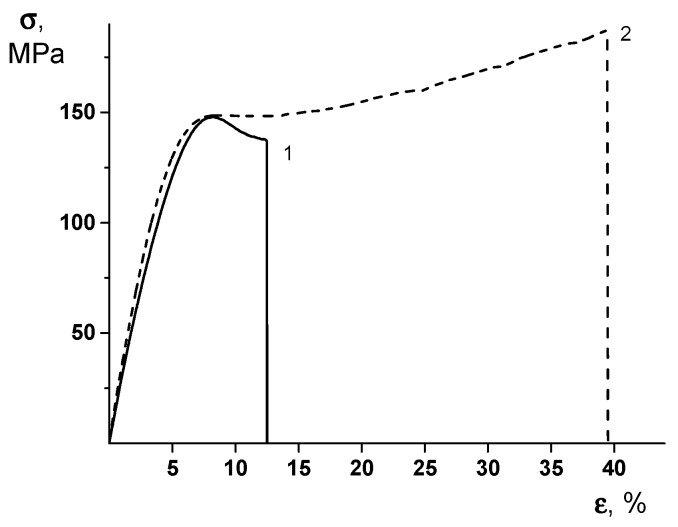
Stress–strain curves of (1) PEA and (2) PEA–Cu(I) membranes.

It is important to note that the formation of the PEA–Cu(I) complex ensures the substantial increase in both the break stress and the ultimate deformation values of the material. To understand the real reason of this unexpected effect, one should examine the stress–strain curves of the PEA and PEA–Cu(I) membranes (Figure 5). The character of the deformation process differs considerably in these materials. In the PEA sample, the predominant mechanism of this process is plastic deformation, with the localization of the deformation via the neck formation after the yield point. Namely, the increased value of the local stress at the point of the necking originates from the film’s break.

The stress–strain curve of the PEA–Cu(I) (Figure 5, curve 2) demonstrates the uniform character of the deformation without the necking process and excessive local strains. This character of the stretching process ensures the increased value of the ultimate deformation of the PEA–Cu(I) membrane, as compared to that of the PEA and, hence, the increase of the break stress. It can be hypothesized that a system of intermolecular crosslinks in the PEA–Cu(I) ensures the uniform distribution of the mechanical stresses in the material during the deformation process and, hence, an increase of the ultimate mechanical properties.

Two thermally stimulated processes with a substantial decrease in the stiffness are registered in the thermomechanical tests of both membranes (Table 3). In the PEA sample, the inherent temperature of the first process, equal to 163 °C, can be attributed to the glass transition temperature. This value is close to *T_g_* values of other polyamide films of similar compositions that were studied previously [34,52]. The formation of the PEA–Cu(I) complex causes the substantial (as high as 60 °C) increase in *T_g_* value; this effect originates from the sizeable restrictions of the segmental mobility of the polymer chains. 

The temperature of the second thermally stimulated process (305−310 °C) is practically similar for both membranes. This transition can be presumably attributed to the melting process in the films. The similarity of the appropriate temperatures of the both films may indicate that the thermal destruction of the complex had already happened in this temperature range and both samples are of the same composition beyond 300 °C.

**Figure 6:**
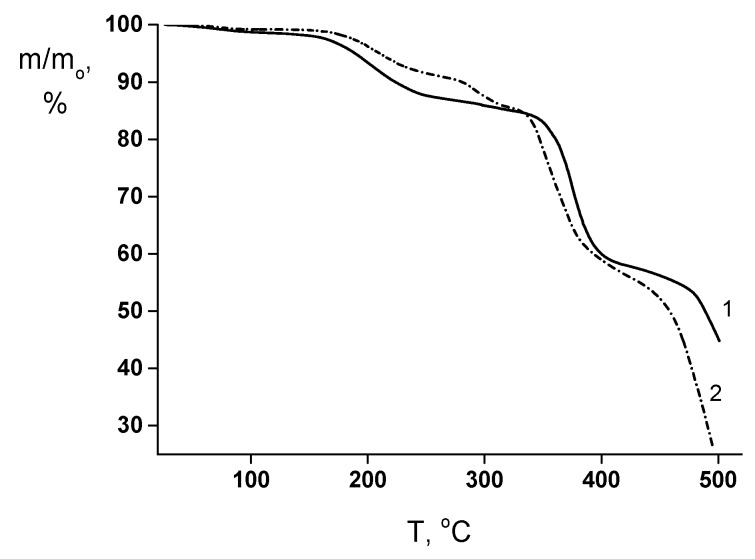
TGA curves of (1) PEA and (2) PEA–Cu(I) membranes.

This assumption is in reasonable agreement with the TGA results (Figure 6). The “weight losses vs. temperature” curves of both membranes contain the initial region from 150 up to 240−250 °C that corresponds to the volatilization of the low-molecular weight impurities (mainly, residual solvent). The thermally stimulated destruction processes start at ~320−330 °C in the both films. However, for the PEA–Cu(I) membrane, the additional process of minor weight losses takes place in the temperature range from 260 up to 310 °C, which is connected with the destruction of metal–polymer complex.

It should be mentioned that high values of Young’s modulus (E, up to 3 GPa), yield stress (*σ_y_*, up to 140 MPa), ultimate deformation (*ε_b_*, 13%−41%), and thermal stability (more than 200 °C) made it possible to consider PEA and PEA–Cu(I) membranes as perspective materials for successful practical use.

### 3.4. Transport PROPERTIES

The choice of mixtures for studying the transport properties of PEA and PEA–Cu(I) membranes is determined by the problem of *n*-heptane extraction from its binary mixtures with thiophene and methanol. Table 4 presents some physical parameters of *n*-heptane, thiophene, and methanol, such as molecular weight (MW), density, volume, boiling temperature (***T_b_***), and dipole moment. It can be seen that *n*-heptane and thiophene have close boiling points, which complicates their separation by the standard distillation methods. It was mentioned above that another pair of liquids, *n*-heptane and methanol, forms the azeotropic mixture [26]. Therefore, the separation *n*-heptane/thiophene and *n*-heptane/methanol mixtures was studied in pervaporation by using PEA and PEA–Cu(I) membranes.

In pervaporation, the mass transfer of liquid molecules through a polymer membrane proceeds according to the following mechanism: sorption of molecules on the membrane surface, their dissolution in the polymer membrane, diffusion through the membrane, and desorption from the other membrane surface. Accordingly, the equilibrium sorption degree (*S*) of liquids was previously determined. 

Sorption studies were carried out by immersion of membrane samples in an individual liquid (*n*-heptane, thiophene, and methanol). Table 5 shows that *n*-heptane is much better adsorbed in the PEA–Cu(I) membrane than in the PEA. The increased value *S* of *n*-heptane for PEA–Cu(I) can be explained by the fact that polymers with complex chain flexibility better absorb nonpolar liquids [53], and the dipole moment (*μ*) of *n*-heptane is equal to 0.

The sorption degree of more polar thiophene molecules for PEA exceeds that for PEA–Cu(I). Probably, coordination of the centers that are responsible for the sorption of thiophene occurs during the creation of metal–polymer complex; as a result, the PEA–Cu(I) membrane exhibits a noticeably lower affinity toward thiophene.

A high sorption degree of methanol was observed for both PEA and PEA–Cu(I) membranes (Table 5); polar methanol molecules (μ = 1.65 D) are well sorbed and penetrate into the free volume elements of both polymer membranes due to their small size.

During sorption and pervaporation, the polymer active centers can interact with liquids (penetrants) by Van der Waals, dipole–dipole, and ion–dipole forces, or by hydrogen-bonding [54]. The interaction of polymer–penetrant is determined by Flory–Huggins parameter (*χ’*), which is calculated by Equation (5). Usually, a stronger interaction between polymer and penetrant would result in a smaller value of *χ’* because the amount of penetrant inside the polymer becomes higher [55]. As seen from Table 5, the lower value of *χ’* is attributed to methanol for both membranes. The highest *χ’* parameters were found for *n*-heptane‒PEA and thiophene‒PEA–Cu(I) pairs; it means that interaction between the polymer and the solvent is weak in these cases.

#### 3.4.1. Pervaporation of Thiophene/n-Heptane Mixture

**Figure 7:**
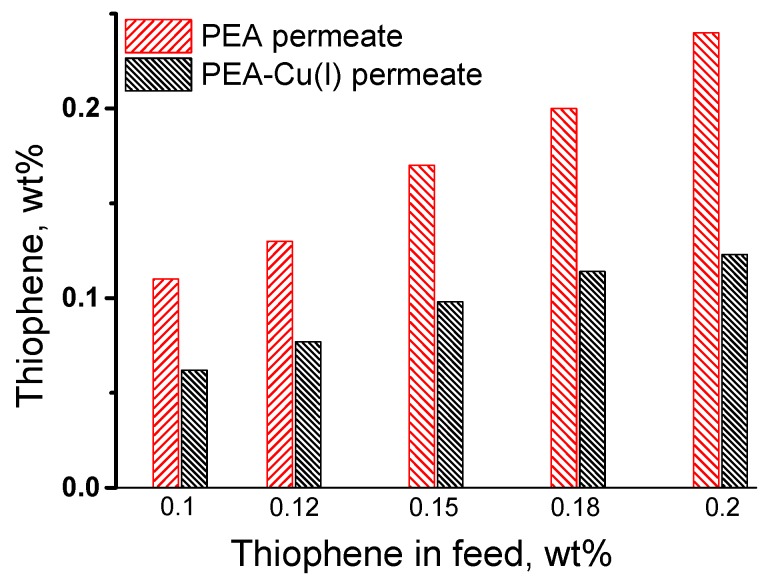
Comparison of the thiophene concentration in the feed and in the permeate after pervaporation of the thiophene/*n*-heptane using PEA and PEA–Cu (I) membranes, 20 °C.

The pervaporation of the thiophene/n-heptane mixture was studied in the range of very low thiophene concentrations, from 0.1 to 0.2 wt%, because the industrial task is purification of oil and fuel from small amounts of sulfur-containing impurities. Figure 7 shows the change in the initial thiophene concentration after pervaporation of the thiophene/n-heptane mixture, using PEA and PEA–Cu(I) membranes. The thiophene concentration in permeate increases compared to its concentration in the feed when using PEA membrane. On the contrary, enrichment of permeate with thiophene does not occur in the case of PEA–Cu(I) membrane. Probably, this result is associated with the sorption activity of the studied membranes, namely, a large affinity of thiophene to the PEA membrane in contrast to the PEA–Cu(I) membrane.

**Figure 8:**
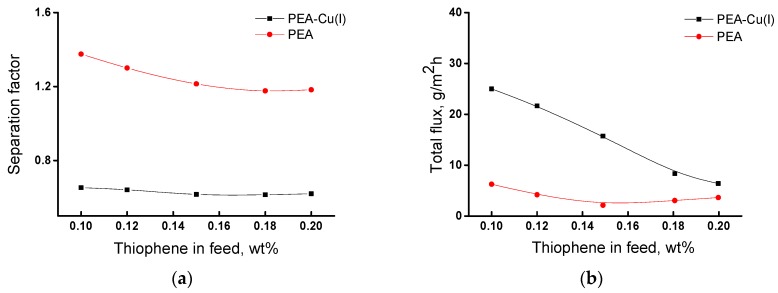
Dependence of (**a**) separation factor α_thiophene/*n*-heptane_ and (**b**) total flux of the membranes on the thiophene concentration in the feed, 20 °C.

Figure 8 shows dependences of the main transport properties of the membranes (separation factor and total flux) on thiophene concentration in the feed in pervaporation of the thiophene/n-heptane mixture. The separation factor changes insignificantly with an increase in thiophene concentration from 0.10 to 0.20 wt% (Figure 8a). For the PEA membrane, the value of separation factor (α) is equal to 1.2–1.4, and for the PEA–Cu(I) membrane α ~0.6, i.e., less than 1. This fact indicates inverse selectivity and preferred permeability of n-heptane through the PEA–Cu(I) membrane.

Figure 8b shows that total flux through the PEA–Cu(I) membrane is larger as compared with the PEA membrane. This may be due to the stronger swelling of the PEA–Cu(I) membrane in the feed containing ~ 99.8 wt% n-heptane, which ensures better formation of transport channels. In addition, the increased free volume in the PEA–Cu(I) structure promotes the permeability of penetrant molecules.

Thus, the study of the transport parameters of the membranes in pervaporation of the thiophene/n-heptane mixture showed that the PEA membrane is more selective for the isolation of thiophene impurities, although the polymer–metal complex exhibits a higher total flux. The membranes under study demonstrate moderate separation properties over those of the literature data [15,16,17,18,21]; however, they are very thermally and mechanically strong and show long-term operational stability.

#### 3.4.2. Pervaporation of Methanol/n-Heptane Mixture

**Figure 9:**
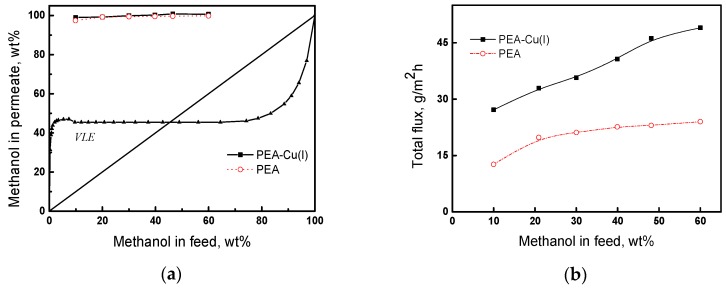
(**a**) Dependence of methanol concentration in the permeate on methanol concentration in the feed for the pervaporation and vapor–liquid equilibrium curve (VLE) of methanol and *n*-heptane mixture; (**b**) dependence of total flux on the methanol concentration in feed, 20 °C.

The pervaporation of the methanol/n-heptane mixture was studied in the range of methanol concentrations from 10 to 60 wt%, at 20 °C. Figure 9a presents the results of pervaporation, as well as the vapor–liquid equilibrium (VLE) curve [56] for the comparison of pervaporation with simple distillation. In pervaporation using both PEA and PEA–Cu(I) membranes, permeate containing more than 95 wt% methanol is formed. The pervaporation separation curves are located much higher than the vapor–liquid equilibrium curve, attesting to the effectiveness of the pervaporation using our membranes. The predominant transport of methanol through the membranes is caused by greater sorption activity of the membranes to methanol, as compared to n-heptane, and greater diffusion ability of smaller methanol molecules, in contrast to that of n-heptane. Since the degrees of methanol sorption are comparable for both the membranes (Table 5), the permeate compositions are almost identical for PEA and PEA–Cu (I).

Figure 9b shows the dependence of the total flux through PEA and PEA–Cu(I) membranes on methanol concentration in the feed. As the methanol concentration increases, the total flux increases, and the flux through the PEA–Cu(I) membrane is about two-times higher than that of PEA. This fact can be attributed to a higher degree of n-heptane sorption in the PEA–Cu(I) membrane than that in the PEA (Table 5), which provides better formation and operation of transport channels, mainly passing the mixture enriched in small methanol molecules.

**Figure 10:**
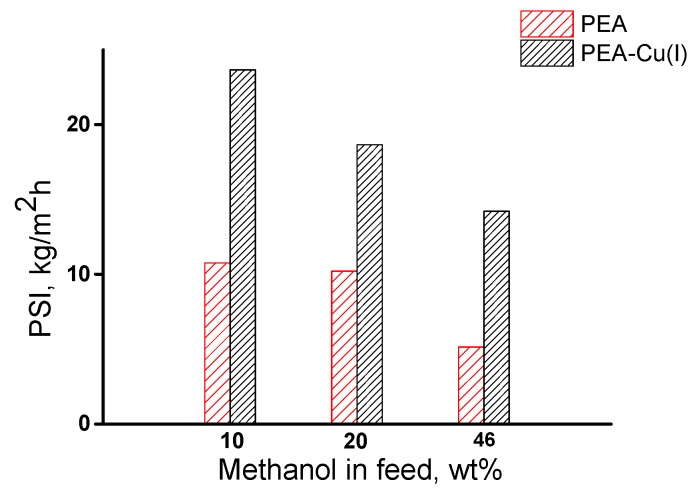
Dependence of pervaporation separation index on methanol concentration in the feed for РЕА and РЕА–Cu(I) membranes, 20 °C;**.**

The overall membrane efficiency was estimated by using the pervaporation separation index (PSI). Figure 10 shows data on PSI for three compositions of methanol/n-heptane mixture, including azeotropic point (46.1 wt% methanol). The РЕА–Cu(I) membrane exhibits significantly higher PSI, as compared with that of РЕА–Cu(I) membrane and allows for highly selective separation of methanol from n-heptane. 

Thus, it was found that both membranes effectively extract methanol from its mixture with *n*-heptane. The pervaporation separation index of the PEA–Cu(I) membrane is much higher than that of the PEA. 

### 3.5. Quantum Mechanical Calculations 

**Figure 11:**
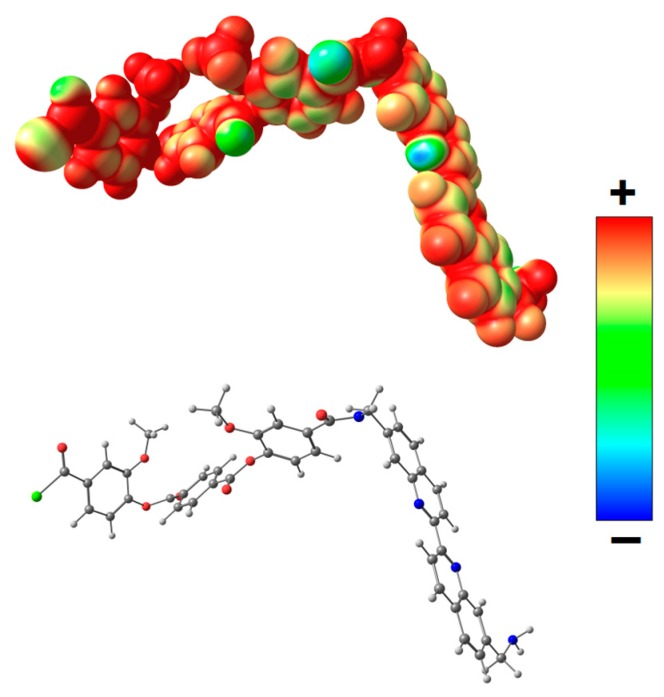
Distribution of electrostatic potential *V_S_*(***r***) calculated on the 0.001 a.u. molecular surface for the optimized equilibrium model structure A. From red to blue, the electrostatic potential is becoming increasingly negative.

For a more detailed investigation of the effect of the complex formed as a result of coordination of biquinoline fragments of PEA with Cu(I) on the transport properties, quantum mechanical calculations were used. Geometry of the model system A (Figure 11) containing biquinoline fragments was optimized, and distribution of molecular electrostatic potential (*V_S_*(***r***)) in this system was calculated. The *V_S_*(***r***) is a function measures the electrostatic interaction between a unit point charge placed at r and the system of interest. It was shown that the most negative electrostatic potential is fixed on nitrogen atoms of biquinoline fragments.

Thus, the screening of nitrogen atoms in PEA by Cu(I) during the formation of metal–polymer complex leads to a lower retention capacity of methanol (and thiophene), that is, the diffusion of methanol (and thiophene) is facilitated through the polymer membrane. This fact determined higher permeability of the PEA–Cu(I) membrane as compared with the PEA. 

## 4. Conclusions

In this study, new polyheteroarylenes with increased chain flexibility, containing ester groups and biquinoline units in the backbone, namely polyester amide PEA and its metal–polymer complex PEA–Cu(I), were synthesized and then used to form the dense flat membranes. According to SEM, the dense homogeneous structure of PEA differs from the morphology of PEA–Cu(I), where small elements of a supramolecular structure with a diameter up to 300 nm appear. The density of PEA increases after complexing with the metal, while the fractional free volume also increases. The PEA–Cu(I) contains both free volume elements caused by loose packing of the polymer chains and additional free volume elements fixed in the polymer matrix due to the loss of fragmentary mobility as a result of coordination around Cu(I). The surface of the PEA–Cu(I) membrane is more hydrophobic compared to PEA. Both polymer membranes are characterized by a reasonably high thermal stability, and the destruction process develops in both materials in the temperature range beyond 300 °C.

Transport properties of PEA and PEA–Cu(I) membranes were studied with the purpose to solve the problem of n-heptane extraction from its binary mixtures with thiophene and methanol. The pervaporation of the thiophene/n-heptane mixture was studied in the range of extremely low thiophene concentrations from 0.1 to 0.2 wt%. It was shown that only the PEA membrane is selective for the separation of thiophene impurities, although the total flux through the PEA–Cu (I) membrane is higher. The study on transport properties during the pervaporation of the methanol/n-heptane mixture in the range of methanol concentrations from 10 to 60 wt% (including mixtures of azeotropic composition) showed that both membranes efficiently remove methanol, and permeate contains more than 95 wt% methanol in all cases. The РЕА–Cu(I) membrane exhibits significantly higher pervaporation separation index as compared with that of РЕА membrane.

The peculiarities of the PEA–Cu (I) transport properties are associated with the fact that, when creating the polymer–metal complex, nitrogen atoms of the biquinoline groups are screened due to coordination with Cu(I), which facilitates the transport of polar methanol molecules. In addition, coordination of macromolecules with Cu(I) impedes the mobility and flexibility of the PEA–Cu(I) chain, which increases the sorption of n-heptane and contributes to the formation of additional transport channels in the swollen membrane. This fact determines the increased total flux through the PEA–Cu(I) membrane during the pervaporation of both methanol/n-heptane and thiophene/n-heptane mixtures.

## Figures and Tables

**Table 1:** Data on density and free volume parameters.

Membrane	Density, g/cm^3^	*V_0_*, cm^3^/g	*V_w_*, cm^3^/g	*V_f_*, cm^3^/g	*FFV*, %
PEA	1.322 ± 0.002	0.756	0.530	0.067	8.9
PEA–Cu(I)	1.329 ± 0.001	0.752	0.526	0.068	9.1

**Table 2:** Contact angles and surface tension of membranes.

Membrane	Contact Angle, °		Surface Tension, mN/m
	Water	Ethanol	
PEA	71.0	18.1	32.2
PEA–Cu(I)	71.3	20.3	31.6

**Table 3:** Mechanical and thermal properties of membranes.

Membrane	*E*, GPa	*σ_y_*, MPa	*σ_b_*, MPa	*ε_b_*, %	Transition Temperatures, °C
PEA	3.06 ± 0.18	147 ± 5	136 ± 5	13 ± 1	163, 305
PEA–Cu(I)	3.38 ± 0.18	146 ± 5	184 ± 5	41 ± 3	223, 310

**Table 4:** Physical properties of the studied liquids.

Liquid	MW	Density, g/cm^3^	Volume, cm^3^/mol	*T_b_*, °C	Dipole Moment, D
*n*-Heptane	100.2	0.684	146.5	98.4	0.00
Thiophene	84.1	1.064	79.0	84.2	0.55
Methanol	32.0	0.792	40.4	64.7	1.65

**Table 5:** Sorption degrees and interaction parameters, *χ′*, for polymer membranes and components of liquid mixtures.

Membrane	*n*-Heptane		Thiophene		Methanol	
	*S*, %	*χ′*	*S*, %	*χ′*	*S*, %	*χ′*
PEA	6.3	1.7	13.0	1.5	20.2	1.13
PEA–Cu(I)	11.1	1.3	9.5	1.7	20.5	1.12
